# Genomic Multiplication and Drug Efflux Influence Ketoconazole Resistance in *Malassezia restricta*

**DOI:** 10.3389/fcimb.2020.00191

**Published:** 2020-04-30

**Authors:** Minji Park, Yong-Joon Cho, Yang Won Lee, Won Hee Jung

**Affiliations:** ^1^Department of Systems Biotechnology, Chung-Ang University, Anseong, South Korea; ^2^School of Biological Sciences and Research Institute of Basic Sciences, Seoul National University, Seoul, South Korea; ^3^Department of Dermatology, School of Medicine, Konkuk University, Seoul, South Korea; ^4^Research Institute of Medicine, Konkuk University, Seoul, South Korea

**Keywords:** *Malassezia restricta*, ketoconazole, resistance, genomic multiplication, efflux pump

## Abstract

*Malassezia restricta* is an opportunistic fungal pathogen on human skin; it is associated with various skin diseases, including seborrheic dermatitis and dandruff, which are usually treated using ketoconazole. In this study, we clinically isolated ketoconazole-resistant *M. restricta* strains (KCTC 27529 and KCTC 27550) from patients with dandruff. To understand the mechanisms of ketoconazole resistance in the isolates, their genomes were sequenced and compared with the susceptible reference strain *M. restricta* KCTC 27527. Using comparative genome analysis, we identified tandem multiplications of the genomic loci containing *ATM1* and *ERG11* homologs in *M. restricta* KCTC 27529 and KCTC 27550, respectively. Additionally, we found that the copy number increase of *ATM1* and *ERG11* is reflected in the increased expression of these genes; moreover, we observed that overexpression of these homologs caused ketoconazole resistance in a genetically tractable fungal pathogen, *Cryptococcus neoformans*. In addition to tandem multiplications of the genomic region containing the *ATM1* homolog, the *PDR5* homolog, which encodes the drug efflux pump protein was upregulated in *M. restricta* KCTC 27529 compared to the reference strain. Biochemical analysis confirmed that drug efflux was highly activated in *M. restricta* KCTC 27529, implying that upregulation of the *PDR5* homolog may also contribute to ketoconazole resistance in the strain. Overall, our results suggest that multiplication of the genomic loci encoding genes involved in ergosterol synthesis, mitochondrial iron metabolism, and oxidative stress response and overexpression of the drug efflux pumps are the mechanisms underlying ketoconazole resistance in *M. restricta*.

## Introduction

The lipophilic yeast *Malassezia restricta* is the most commonly found fungus on human skin; it is implicated in skin diseases such as seborrheic dermatitis and dandruff (Clavaud et al., 2013; Findley et al., 2013; Xu et al., 2016; Park T. et al., 2017). Multiple tropical drugs with antifungal activity against *Malassezia* have been used for the treatment of skin diseases associated with *M. restricta* (Carrillo-Muñoz et al., 2013; Cafarchia et al., 2015; Rojas et al., 2017). Among these antifungal drugs, ketoconazole, an imidazole compound, exhibits highly effective fungistatic activity against *Malassezia*; the effectiveness of this azole against seborrheic dermatitis and dandruff has also been demonstrated (Danby et al., 1993; Pierard-Franchimont et al., 2001, 2002).

Azole antifungal drugs, including ketoconazole, inhibit a cytochrome P450 enzyme, lanosterol 14α-demethylase, which participates in the synthesis of ergosterol, a major constituent of the fungal cell membrane (Vanden Bossche et al., 1987; Yoshida and Aoyama, 1987). Inactivation of this enzyme results in the demethylation of lanosterol, which inhibits the process of ergosterol synthesis, leading to accumulation of the bypass product as toxic methylated sterols in the fungal cell membrane, defects in fungal plasma membrane integrity, and inhibition of cell growth (Joseph-Horne and Hollomon, 1997; Heimark et al., 2002). However, as the azole drug is fungistatic, prolonged use of antifungals offers the opportunity for pathogenic fungi to acquire drug resistance. For example, since the first reports of the emergence of miconazole-resistant *Candida albicans* in 1978, clinical isolates of azole-resistant *Candida* species have been identified continuously (Holt and Azmi, 1978; Ksiezopolska and Gabaldón, 2018). *Cryptococcus* and *Aspergillus* isolates that are resistant to azole antifungal drugs have also been reported frequently (Smith et al., 2015; Rivero-Menendez et al., 2016).

Azole resistance mechanisms involve the *ERG11* gene encoding a lanosterol 14α-demethylase, which is the direct target enzyme of azole antifungal drugs. Mutations in the coding region of the gene result in amino acid substitutions, which alter the structure of the enzyme and reduce the affinity of the target for the azole (Sanglard et al., 1998; Warrilow et al., 2010). Overexpression of *ERG11*, resulting in increased levels of the antifungal target protein, is also considered one of the mechanisms of azole resistance in fungi (Flowers et al., 2012; Feng et al., 2017).

Besides mutations and overexpression of *ERG11*, increased drug efflux resulting in decreased intracellular drug accumulation is known to be one of the major mechanisms of azole resistance. In *C. albicans*, two efflux pumps of the ATP-binding cassette (ABC) transporter family, Cdr1 (*Candida* drug resistance (1) and Cdr2 are well known to be linked with resistance to azole antifungal drugs (Prasad et al., 1995; Sanglard et al., 1997). Further, a number of azole-resistant *C. albicans* isolates showed overexpression of *CDR1* and *CDR2*; deletion of one or both genes resulted in hyper-susceptibility to azoles (White, 1997; Lyons and White, 2000; Tsao et al., 2009). Another efflux protein, Mdr1, a transporter of the major facilitator superfamily (MFS) class is involved in fluconazole resistance; this gene was found to be overexpressed in fluconazole-resistant *C. albicans* isolates (White, 1997; Lyons and White, 2000; Hiller et al., 2006; Feng et al., 2018).

Most *Malassezia* strains are sensitive to azole drugs; however, recent studies reported the emergence of azole-resistant *Malassezia* species (Jesus et al., 2011; Nijima et al., 2011; Cafarchia et al., 2012a,b; Iatta et al., 2014; Kim et al., 2018). To date, two studies have investigated the mechanisms of azole susceptibility and resistance in *Malassezia*. Iatta et al. suggested that the drug efflux pump is involved in azole resistance in *M. pachydermatis* and *M. furfur* (Iatta et al., 2017); Kim et al. demonstrated that tandem quadruplication of the genomic region containing the genes required for ergosterol synthesis contributes to azole resistance in *M. pachydermatis* (Kim et al., 2018). However, these studies were mainly focused on *M. pachydermatis* residing on canine skin, and no study, to our knowledge, has reported the isolation of azole-resistant *M. restricta*, the most predominant *Malassezia* species on human skin, and analyzed the mechanism of its resistance.

Thus, in this study, we isolated ketoconazole-resistant *M. restricta* strains from dandruff patients and aimed to elucidate their resistance mechanisms using comparative genome analysis. In the resistant isolates, we found a tandemly multiplicated genomic locus along with increased gene expression and hypothesized that the genomic multiplication contributes to azole resistance in *M. restricta*. Further, we observed increased drug efflux in a resistant isolate, suggesting that this also influences resistance in *M. restricta*.

## Materials and Methods

### Strains and Growth Conditions

*Malassezia restricta* strains were grown on LNA medium (0.5% glucose, 1% peptone, 0.01% yeast extract, 0.8% bile salt, 0.1% glycerol, 0.05% glycerol monostearate, 0.05% Tween 60, 1.2% agar, and 0.5% whole fat cow milk) at 34°C for 3 days (Leeming and Notman, 1987; Guillot and Gueho, 1995; Park M. et al., 2017). The wild type *Cryptococcus neoformans* var. *grubii* H99 and the overexpression strain were grown in YPD medium (1% yeast extract, 2% peptone, and 2% glucose) or YPG medium (1% yeast extract, 2% peptone, and 2% galactose) at 30°C for 1–2 days.

### Drug Susceptibility Tests

Minimal inhibitory concentrations (MICs) were determined using the method described by Gupta et al. and Sugita et al. with slight modifications (Gupta et al., 2000; Sugita et al., 2005). Briefly, 20 μL of antifungal drug compounds (50 × of stock solution) were serially diluted 2-fold with 980 μL of melted LNA medium in a 24-well plate. Ketoconazole, terbinafine, amphotericin B, and zinc pyrithione (ZPT) were used to test the drug susceptibility of *M. restricta*. The yeast cells were inoculated into each well of the 24-well plate and incubated at 34°C for 3 days. The MIC values were determined as the lowest concentration at which growth was invisible compared with that in the medium without any drugs. To estimate the ketoconazole susceptibility of the *C. neoformans* strain overexpressing *ATM1* and *ERG11*, 10-fold serial dilutions of cells starting at 10^4^ cells were spotted onto YPD or YPG agar plates with or without ketoconazole and incubated at 30°C for 2 days. To evaluate the susceptibility of oxidative stress in *M. restricta* strains, ten-fold serial dilutions of cells starting at 10^7^ cells were spotted onto LNA plates with or without H_2_O_2_. Plates were incubated at 34°C for 7 days.

### Genome Sequencing

*Malassezia restricta* KCTC 27529 and KCTC 27550 were grown on LNA medium at 34°C for 3 days; genomic DNA from the cells was extracted using glass beads and vortexing as described previously (Van Burik et al., 1998). Genome sequencing of *M. restricta* KCTC 27529 and KCTC 27550 was performed using the Illumina MiSeq and PacBio RSII platforms with C4 chemistry. Illumina libraries were constructed using the TruSeq DNA Library Prep LT Kit (Illumina, USA) according to the manufacturer's instructions. The constructed libraries were sequenced on the Illumina MiSeq instrument and 300 bp paired-end reads were generated. Raw reads were quality-trimmed using Trimmomatic v0.36 and mapped to the reference genome using bowtie2 v2.2.5 with the “–very-sensitive” option (Langmead and Salzberg, 2012; Bolger et al., 2014). The coverage of each gene and that of the whole genome were calculated using the length and the number of mapped bases. Finally, the copy number of each gene was determined by dividing its coverage with the average in the entire genome. The PacBio library, with 20-kb inserts, was prepared using the PacBio Sample Net-Shared Protocol (available at http://pacificbiosciences.com/). The constructed libraries were loaded into one SMRT cell for each sample and sequenced using the PacBio RS II instrument (PacBio, Menlo Park, CA, USA). Data from the genome sequencing were deposited in the Sequence Read Archive (SRA) database of the National Center for Biotechnology Information (NCBI) (Bioproject number, PRJNA592379).

### Identification of Single Nucleotide Mutations in *ERG11*

The coding region of *ERG11* from the ketoconazole-susceptible *M. restricta* isolates was amplified by PCR using the primers ERG11_start1 and ERG11-4 (Supplementary Table 1). The resulting amplified PCR products were sequenced using ERG11_start2, ERG11-2, ERG11-3, and ERG11-4. The obtained sequences were compared with the *ERG11* sequence of the reference strain, *M. restricta* KCTC 27527.

### Construction of the Overexpression Strains of *C. neoformans*

To overexpress *ATM1* in *C. neoformans*, the native promoter of the gene was substituted with the *TEF1* promoter via homologous recombination. The *TEF1* promoter was amplified by PCR using *C. neoformans* H99 genomic DNA as a template and the primers TEF1p_F_XbaI and TEF1p_R_XhoI. The PCR product was digested using XbaI and XhoI and subsequently cloned into the plasmid pJAF1 containing the neomycin resistance (*NEO*^*R*^) gene; the resulting plasmid was named pWH132 (Fraser et al., 2003). The DNA fragment containing the *NEO*^*R*^ gene and *TEF1* promoter was amplified by PCR using the plasmid pWH132 as a template and the universal primers M13-F and M13-R. The 5′ flanking region containing the native promoter of *ATM1* and the 3′ flanking region containing the partial coding sequence of the gene from the start codon to 841 bp were amplified by PCR using the primers TEF1p_ATM1-1/TEF1p_ATM1-2 and TEF1p_ATM1-3/TEF1p_ATM1-4, respectively, with H99 genomic DNA as the template. The 5′ and 3′ flanking regions and the *NEO*^*R*^-P_*TEF*1_ region were fused by overlapping PCR using primers TEF1p_ATM1-5 and TEF1p_ATM1-6. The resulting cassette was biolistically transformed into the H99 strain as described previously (Toffaletti et al., 1993). The used primers are listed in Supplementary Table 1. The *ERG11* overexpression strain that was constructed in our previous study was used in the current study (Kim et al., 2012).

### RNA Isolation and cDNA Synthesis

*M. restricta* KCTC 27527, KCTC 27529, and KCTC 27550 were grown on LNA medium at 34°C for 3 days; *C. neoformans* H99 and strains overexpressing *ATM1* and *ERG11* were cultured at 30°C overnight in YPD or YPG medium. Total RNA was extracted from the cells using TransZol Up (TransGen Biotech, China) and used to synthesize cDNA. cDNA was synthesized using the RevertAid First Strand cDNA synthesis Kit (Thermo Fisher Scientific, Waltham, MA, USA) according to the manufacturer's instructions.

### Quantitative Real-Time PCR

To validate the gene copy number and gene expression, quantitative real-time PCR (qRT-PCR) was performed. Genomic DNA and cDNA were used as templates for gene copy number and gene expression, respectively. Gene-specific primers for qRT-PCR were designed using Primer Express software 3.0 (Applied Biosystems, Foster, CA, US) and are listed in Supplementary Table 1. Relative quantitation of gene expression was performed using the 2^Δ^Δ*CT* method on a 7500 system (Applied Biosystems, Foster, CA, US) (Livak and Schmittgen, 2001). The actin gene (MRET_1518) and *TEF2* (CNAG_00044, translation elongation factor 2) were used as endogenous control genes of *M. restricta* and *C. neoformans*, respectively.

### Flow Cytometric Analysis

Cells were grown at 34°C for 3 days, and 2 × 10^7^ cells/mL of each strain were resuspended in 20 mL of modified Dixon's (mDixon's) medium (3.6% malt extract, 0.6% peptone, 2% bile salt, 1% Tween 40, 0.2% oleic acid, and 0.2% glycerol) with or without 10 μM rhodamine 6G (R6G), followed by incubation at 34°C for 2 h (Midgley, 1989). Uptake of R6G into the cells was stopped by cooling the reaction mixture on ice. To determine R6G accumulation, samples were diluted 40-fold in cold 0.1 M phosphate-buffered saline (PBS) and directly subjected to flow cytometry using FACSAria II (BD Bioscience, San Jose, CA, USA) at a wavelength of 488 nm. The data of 5,000 cells from each sample were collected at the PE channel and analyzed using FACS DIVA (BD Bioscience, San Jose, CA, USA). For efflux of R6G, cells were washed twice with cold 0.1 M PBS and suspended in PBS, followed by incubation at 34°C for 30 min. The samples were then prepared and analyzed as described above.

### Statistical Analysis

All data are presented as the arithmetic mean ± standard deviation. Differences between samples were calculated by two-tailed Student's *t*-test for unpaired data. A *p* < 0.01 was considered statistically significant.

## Results

### Isolation of Ketoconazole-Resistant *M. restricta* Strains

Antifungal susceptibility of the clinical isolates of the *M. restricta* strains, which were obtained from Korean patients with severe dandruff previously (Park M. et al., 2017), was evaluated along with the type strain, *M. restricta* CBS 7877. MICs for ketoconazole, terbinafine, amphotericin B, and ZPT were determined as described in the Materials and Methods (Table 1). The MIC values of all *M. restricta* strains, including the type strain, against terbinafine, amphotericin B, and ZPT ranged from 0.25 to 2, 2 to 8, and 0.795 to 3.18 μg/mL, respectively. However, in the case of ketoconazole, two isolates, *M. restricta* KCTC 27529 and KCTC 27550, showed an MIC of 3.89–7.68 μg/mL, which is 16–256 times higher than those of the other *M. restricta* strains, including the type strain CBS 7877, which showed an MIC of 0.03–0.24 μg/mL. These results indicate that *M. restricta* KCTC 27529 and KCTC 27550 are specifically resistant to ketoconazole, an imidazole antifungal drug that inhibits ergosterol biosynthesis in fungi. These strains were thus selected for further investigation of ketoconazole resistance in *M. restricta*.

**Table 1:** MIC values of antifungal agents in *M. restricta*.

**Strain**	**Ketoconazole**	**Terbinafine**	**Amphotericin B**	**ZPT**
KCTC 27512	0.06–0.12	0.25–0.5	4	1.59
KCTC 27518	0.03–0.06	0.5–1	4–8	0.795
KCTC 27519	0.12–0.24	0.5	4–8	3.18
KCTC 27522	0.06–0.12	0.25–0.5	4	3.18
KCTC 27527	0.03	0.5	2	1.59
KCTC 27529	7.68	2	2–4	3.18
KCTC 27539	0.03–0.06	0.5–1	2–4	1.59
KCTC 27540	0.06–0.12	0.5–1	4–8	3.18
KCTC 27542	0.06–0.12	1	4–8	1.59
KCTC 27543	0.06	0.5–1	4–8	3.18
KCTC 27544	0.03–0.06	0.5–1	8	3.18
KCTC 27548	0.12	0.5–1	4–8	1.59
KCTC 27550	7.68	0.5	4	1.59
CBS 7877	0.06–0.12	1–2	4	0.795

*(Unit: μg/mL)*.

### Genome Analysis of the Resistant Strains

To investigate the underlying mechanism of altered ketoconazole sensitivity at the genome-wide level, genomes of *M. restricta* KCTC 27529 and KCTC 27550 were sequenced and analyzed in comparison with the genome of the susceptible reference strain, *M. restricta* KCTC 27527 (Cho et al., 2019). We used *M. restricta* KCTC 27527 as a susceptible reference strain rather than the type strain CBS 7877 because KCTC 27529 and KCTC 27550 are phylogenetically closer to KCTC 27527 than CBS 7877, and belong to the same sub-species level molecular type (Park M. et al., 2017).

The genomes of the resistant strains were sequenced using a combination of Illumina HiSeq and PacBio Sequel technologies, of which the sequencing method was principally used to increase the overall quality of genome sequencing analysis and investigate a possible copy number variation in the resistant strains. Initially, we investigated the distribution of single nucleotide mutations in the resistant strains and found that, compared to the total 7,330,907 bp in the genome of the reference strain KCTC 27527, 35,992 (0.49%) and 37,178 (0.51%) single nucleotide mutations were found in the genomes of the two resistant isolates, KCTC 27529 and KCTC 27550, respectively. Among these, 7,401 (0.10%) and 7,737 (0.11%) single nucleotide mutations resulted in amino acid substitutions in each strain, respectively. The distribution and the nature of the mutations in the genomes of the resistance strains were listed in the Supplementary Figure 1.

### Mutations in the *ERG11* Homolog Were Identified in the Resistant Strains

**Figure 1:**
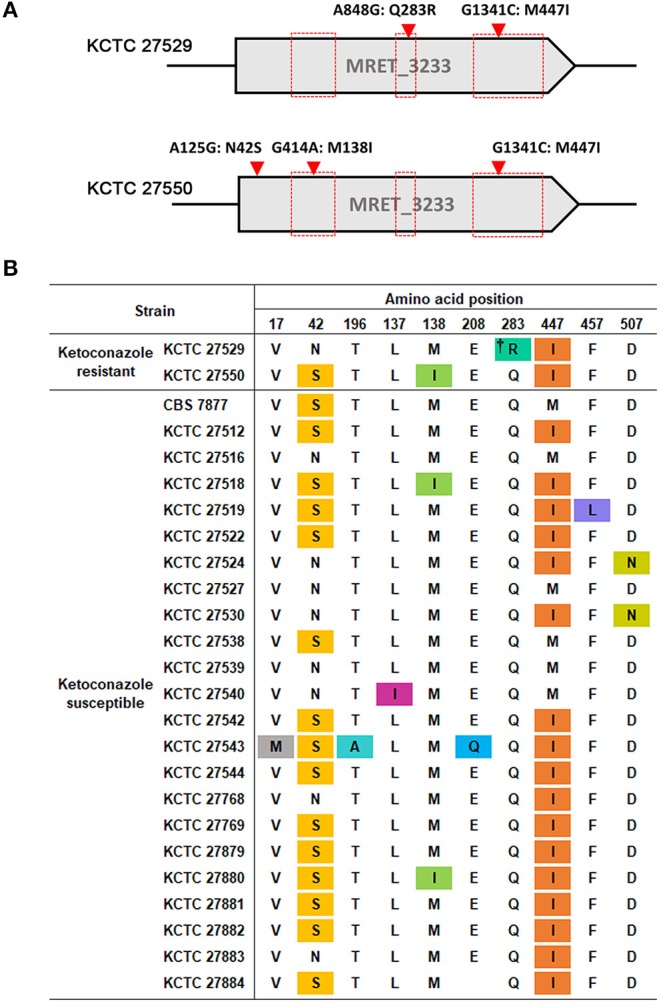
Nonsynonymous substitution mutations in the *ERG11* homolog of *M. restricta* strains. **(A)** Nucleotide and amino acid substitutions in the *ERG11* homolog of ketoconazole-resistant strains, *M. restricta* KCTC 27529 and KCTC 27550, were compared with those of the susceptible strain, *M. restricta* KCTC 27527. Red boxes indicate the hotspot regions at which mutations frequently occur in the azole-resistant *C. albicans* strains (Marichal et al., 1999). **(B)** Amino acid substitutions in the *ERG11* homolog of ketoconazole susceptible *M. restricta* strains compared with that of the reference strain KCTC 27527. The colored amino acids indicate nonsynonymous mutations found in the susceptible *M. restricta* strains. †Q283R was found to be not related to azole resistance in *C. albicans* C507 (Chau et al., 2004).

One of the well-known mechanisms of azole resistance includes nonsynonymous substitution mutations within the coding region of *ERG11*, which reduces the binding affinity of lanosterol 14α-demethylase to azoles (Cowen et al., 2002). One of the examples includes the reduced affinity of the *C. albicans* Erg11 protein possessing G464S or R467K amino acid substitution to fluconazole (Kelly et al., 1999; Lamb et al., 2000). We were, therefore, particularly concerned about single nucleotide mutations in the *ERG11* homolog, MRET 3233, in the resistant strains KCTC 27529 and KCTC 27550. The results of our genome sequencing analysis revealed that KCTC 27529 and KCTC 27550 possess two (A848G:Q283R and G1341C:M447I) and three (A125G:N42S, G414A:M138I, and G1341:M447I) nonsynonymous substitution mutations, respectively, compared to the susceptible reference strain KCTC 27527. Among these mutations, G1341C: M447I was commonly observed in both KCTC 27529 and KCTC 27550 (Figure 1A). In KCTC 27529, all mutations were located within the hotspot regions in which mutations are frequently observed in azole-resistant *C. albicans* (Marichal et al., 1999). In KCTC 27550, all mutations except N42S were located within these regions.

We reasoned that the nonsynonymous substitution mutations observed within the *ERG11* homologs of the resistant strains would not exist in the ketoconazole-susceptible *M. restricta* strain if they influenced ketoconazole resistance. Thus, the nucleotide sequences of the *ERG11* homologs of other ketoconazole-susceptible strains were determined; we found that all mutations, except the Q283R mutation that was identified in the resistant strain KCTC 27529, were present in the *ERG11* homologs of the ketoconazole-susceptible strains (Figure 1B). Therefore, we ruled out the association of nonsynonymous substitution mutations identified in the *ERG11* homolog of the ketoconazole-resistant strains KCTC 27529 and KCTC 27550. The Q283R mutation was also excluded because the same mutation was identified in the fluconazole-resistant *C. albicans* strain C507 and was found not to be related to azole resistance (Chau et al., 2004). In addition, according to available crystal structures of the Erg11 protein, R283 in KCTC 27529 is corresponds to H283, C270, and V232 in *C. albicans, Aspergillus fumigatus*, and *Mycobacterium tuberculosis* respectively, which are located far outside of the heme-containing active site (Podust et al., 2001; Xiao et al., 2004). Normally, amino acid substitutions located in the region surrounding the central heme group disturb the azole binding and confer resistance (Monk et al., 2014; Flowers et al., 2015). Therefore, the effect of Q283R on the resistance of KCTC 27529 might be minimal.

### Tandem Multiplications of Specific Genomic Loci Influence Ketoconazole Resistance

**Figure 2:**
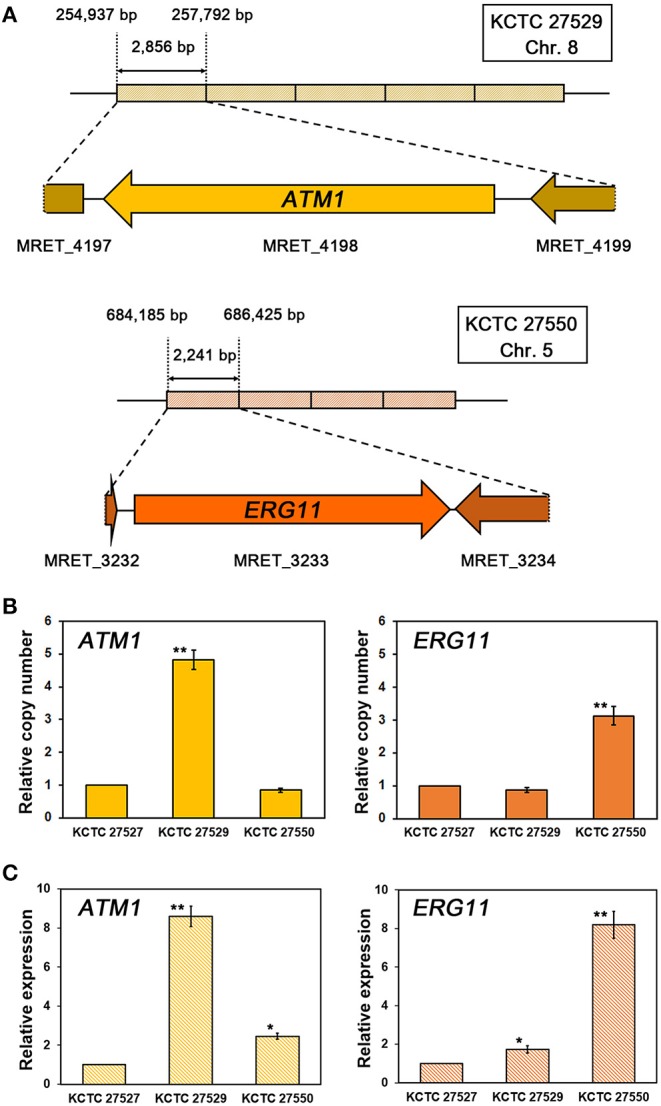
Tandem multiplication of genomic loci and confirmation of gene copy numbers and expression. **(A)** The genomic loci containing MRET_4198, the *ATM1* homolog in chromosome 8 of *M. restricta* KCTC 27529, and MRET_3233, the *ERG11* homolog in chromosome 5 of *M. restricta* KCTC 27550, are tandemly multiplicated five and four times, respectively. The relative copy number **(B)** and expression **(C)** of the *ATM1* and *ERG11* homologs in each resistant strain were confirmed using qRT-PCR. Values were normalized using the actin gene (MRET_1518) as an endogenous control and compared to the susceptible reference strain *M. restricta* KCTC 27527. The results are averages of three biological replicates (*p < 0.01, **p < 0.001).

In addition to the identification of mutations in the *ERG11* homologs, our genome analysis revealed that multiplications of specific genomic loci occurred in the genomes of each resistant strain. We found that, in the resistant strain KCTC 27529, the genomic locus of the 2,856 bp region containing the complete coding region of MRET_4198 on chromosome 8 is tandemly repeated five times. Tandem multiplications of the genomic locus were also identified in the resistant strain KCTC 27550; moreover, the 2,241 bp region containing the complete coding region of MRET_3233 on chromosome 5 was tandemly repeated four times (Figure 2A). The tandem multiplications of the loci in each resistant strain were further confirmed by qRT-PCR using the genomic DNA of each resistant strain as a template; the results revealed a relative increase in the copy number of MRET_4198 and MRET_3233 in KCTC 27529 and KCTC 27550, respectively (Figure 2B). Genomic rearrangements such as multiplication and translocation, have been frequently observed and can lead to phenotypic differences and genome evolution in various eukaryotic organisms; further, the rearrangement event is suggested to be involved in direct or inverted repeat sequences at the boundaries of the genomic region (Koszul et al., 2004; Carvalho et al., 2011; Beck et al., 2015). However, no repeated sequence was identified in the boundaries of multiplicated loci in the genome of KCTC 27529 and KCTC 27550.

While MRET_3233 is a homolog of *ERG11*, MRET_4198 is a homolog of *ATM1* that encodes a mitochondrial inner membrane ATP-binding cassette (ABC) transporter and is required for iron metabolism such as Fe-S cluster biogenesis and heme synthesis (Kispal et al., 1997, 1999). To confirm that multiplication of the *ATM1* and *ERG11* homolog genes increased the transcript levels of each gene, the gene expression levels were determined; the results revealed significant upregulation of each gene in the resistant strain compared to the susceptible reference strain, KCTC 27527 (8.60 ± 0.52 and 8.19 ± 0.70-fold increase, respectively) (Figure 2C).

**Figure 3:**
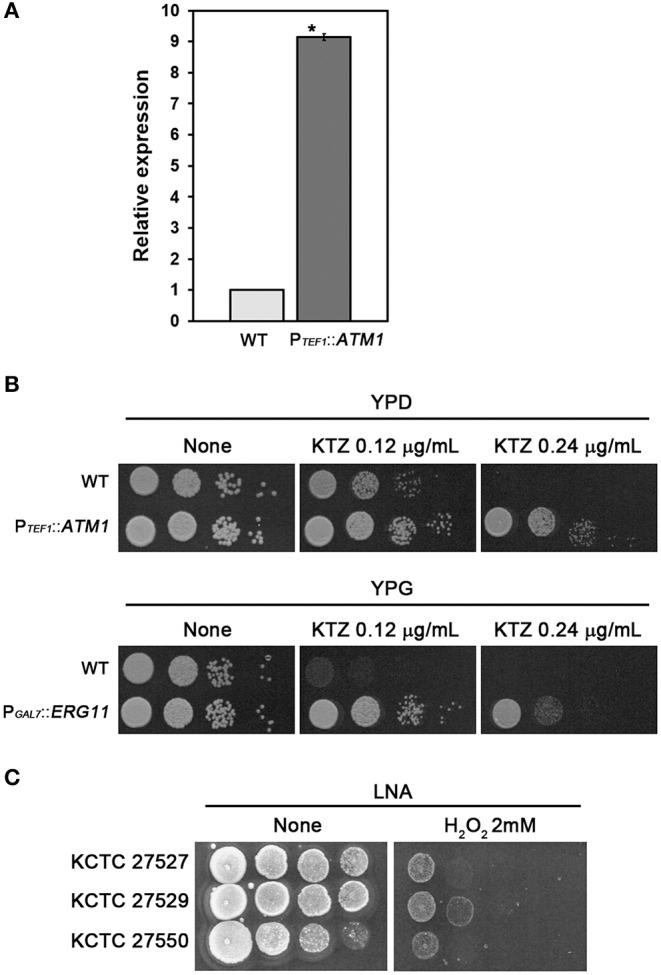
Contribution of *ATM1* and *ERG11* overexpression to ketoconazole resistance. **(A)** Overexpression of *ATM1* in *C. neoformans* was confirmed using qRT-PCR. Values were normalized using *TEF2* as an endogenous control and compared to the wild type strain. The results are averages of three biological replicates (*p < 0.001). **(B)** The growth of *C. neoformans* overexpressing *ATM1* in YPD media containing ketoconazole was monitored (upper panel). The growth of the *C. neoformans* strain overexpressing *ERG11* in YPG media containing ketoconazole was monitored (lower panel). The *C. neoformans* strain harboring P_*GAL*7_::*ERG11*, which was constructed and confirmed in our previous study, was used (Kim et al., 2012). Cells were serially diluted 10-fold, spotted onto the plate, and incubated at 30°C for 2 days. WT, wild type; P_*TEF*1_::*ATM1*, overexpression of *ATM1*; P_*GAL*7_::*ERG11*, overexpression of *ERG11*. **(C)** The growth of *M. restricta* KCTC 27527, KCTC 27529, and KCTC 27550 in LNA media containing hydrogen peroxide was monitored. Cells were serially diluted 10-fold, spotted onto the plate, and incubated at 34°C for 7 days.

We next investigated whether upregulation of the tandemly multiplicated *ATM1* and *ERG11* homologs contributed to azole resistance in the ketoconazole-resistant strains KCTC 27529 and KCTC 27550. As no genetic manipulation tools have been developed yet for *M. restricta*, we utilized a genetically tractable model pathogenic fungus, *Cryptococcus neoformans* (Hull and Heitman, 2002). *ATM1* was fused with the *TEF1* promoter and integrated into its authentic locus in the genome of *C. neoformans*. Overexpression of *ATM1* in *C. neoformans* was confirmed by qRT-PCR; subsequently, the growth of fungal cells was challenged in the presence of ketoconazole (Figures 3A,B). The results of growth analysis showed that *ATM1* overexpression significantly reduced the sensitivity of fungal cells to ketoconazole, implying that the increased gene expression contributes to ketoconazole resistance in *M. restricta* KCTC 27529. Previously, we constructed the *C. neoformans* strain overexpressing *ERG11* under the *GAL7* promoter and demonstrated that the overexpression of gene homologs increases resistance to the azole antifungal drug, fluconazole (Kim et al., 2012). In the current study, we used the same overexpression strain and confirmed that *ERG11* overexpression reduced the sensitivity of fungal cells to ketoconazole, implying that increased expression of the gene contributes to ketoconazole resistance in *M. restricta* KCTC 27550 (Figure 3B).

Lack of *ATM1* in *S. cerevisiae* and *C. neoformans* induces hypersensitivity to hydrogen peroxide, which generates oxygen-derived free radicals in the cell, indicating that Atm1 is involved in the defense mechanism against oxidative stress in the fungal cells (Kispal et al., 1997; Do et al., 2018), and a study showed that ketoconazole generated reactive oxygen species in *C. albicans* (Snell et al., 2012). Therefore, we hypothesized that the resistance strain KCTC 27529 would show reduced sensitivity to oxidative stress and evaluated sensitivity of the strains to hydrogen peroxide. The results showed that *M. restricta* KCTC 27529 is less sensitive to hydrogen peroxide confirming the possible association between the increased expression of *ATM1* and ketoconazole resistance in the (Figure 3C). Collectively, our results suggest that the increased expression of *ATM1* and *ERG11* homologs caused by genomic tandem multiplication of each locus is one of the main causes of ketoconazole resistance in *M. restricta* KCTC 27529 and KCTC 27550.

### Altered Expression of the *PDR5* Homolog Influences Ketoconazole Resistance

**Figure 4:**
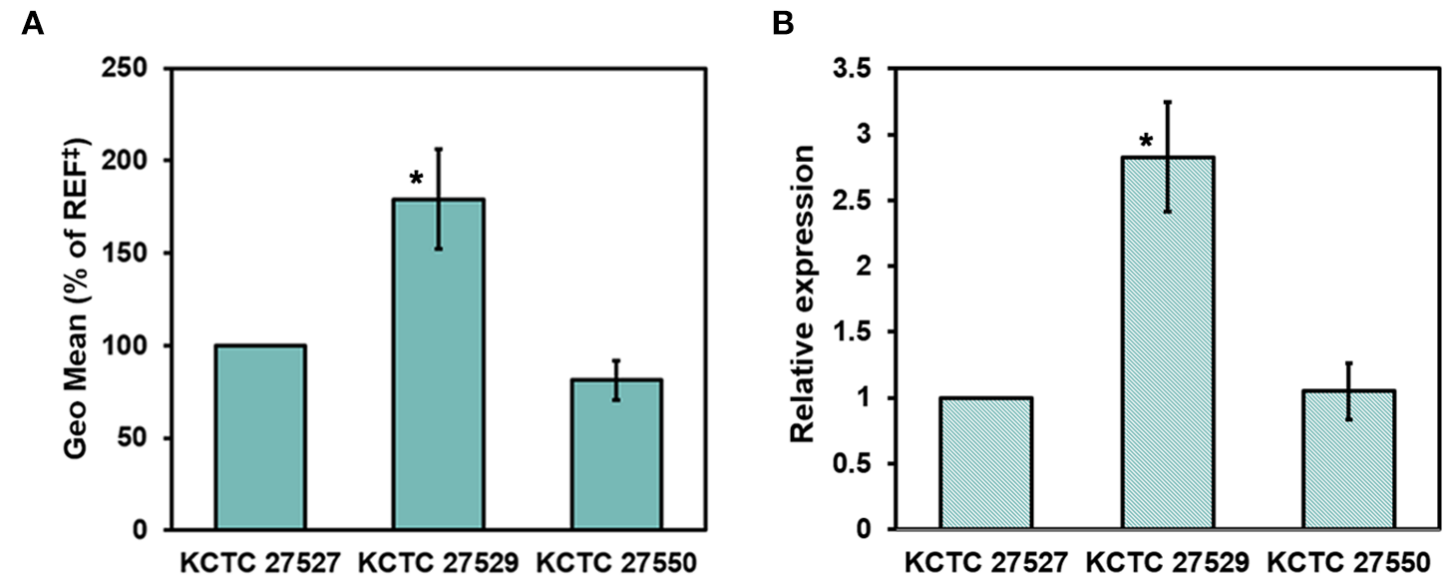
Evaluation of drug efflux in *Malassezia restricta*. **(A)** The geometric means of differences between the efflux and accumulation of R6G in *M. restricta* KCTC 27529 and KCTC 27550 were compared to the reference strain, KCTC 27527. Values were compared to the reference strain KCTC 27527. The results are averages of three biological replicates (*p < 0.005). **(B)** Relative expression of the *PDR5* homolog was determined by qRT-PCR. Values were normalized using the actin gene (MRET_1518) as an endogenous control and compared to the reference strain KCTC 27527. The results are averages of three biological replicates (*p < 0.005). ^‡^REF indicates the reference strain *M. restricta* KCTC 27527.

Hyperactivation of drug efflux reduces the intracellular accumulation of azoles and has been considered one of the major drug resistance mechanisms in pathogenic fungi (Morio et al., 2017). This phenomenon led us to investigate drug efflux in the resistant strains KCTC 27529 and KCTC 27550 to find an additional resistance mechanism that may exist in ketoconazole-resistant strains. Drug efflux in *M. restricta* cells was determined using rhodamine 6G (R6G) (Maesaki et al., 1999); the results revealed that drug efflux of the resistant strain KCTC 27529 was significantly increased compared with that of the susceptible reference strain KCTC 27527, whereas the drug efflux of the resistant strain KCTC 27550 was similar to that of the reference strain (Figure 4A). Furthermore, addition of the efflux pump inhibitor promethazine (PMZ) reduced the MIC values of KCTC 27529 significantly (Iatta et al., 2017) (Table 2). These results suggested that increased drug efflux also contributes to ketoconazole resistance in KCTC 27529.

**Table 2:** MIC values of ketoconazole with efflux inhibitor promethazine (PMZ).

	**KCTC 27527**	**KCTC 27529**	**KCTC 27550**
Ketoconazole	0.03–0.06	3.89-7.68	3.89-7.68
Ketoconazole + promethazine 50 μg/mL	0.03–0.06	0.98	3.89-7.68
Ketoconazole + promethazine 100 μg/mL	0.06	0.48	3.89-7.68

*(Unit: μg/mL)*.

To further confirm the increased drug efflux in KCTC 27529, we searched the genome for the homolog of *S. cerevisiae* Pdr5, which is involved in drug efflux, including the efflux of azole antifungal drugs (Paul and Moye-Rowley, 2014; Morio et al., 2017), analyzed its expression levels, and compared them with those in the susceptible strain. MRET_2329 was found to be the only homolog of *S. cerevisiae* Pdr5 (38.36% identity) in *M. restricta*; its expression was considerably increased (2.83 ± 0.41-fold) in the ketoconazole-resistant strain KCTC 27529 compared to the susceptible reference strain (Figure 4B). In *C. albicans*, an additional ABC transporter, Cdr2, plays a role in azole drug resistance along with Cdr1, the homolog of *S. cerevisiae* Pdr5 (Sanglard et al., 1997). We found that *M. restricta* possesses the homolog of *C. albicans* Cdr2, MRET_2330 (38.32% identity). However, the expression levels of the Cdr2 homolog in *M. restricta* were similar to those in the susceptible reference strain. Flr1 is involved in efflux of fluconazole in *S. cerevisiae*, and we identified the homolog in *M. restricta*, MRET_3736 (33% identity). However, the expression levels of the Flr1 homolog were also similar to that in the reference strain. These results suggested that increased drug efflux in KCTC 27529 was solely caused by hyperactivation of the *PDR5* gene MRET_2329. Collectively, our results suggested that increased drug efflux, along with genomic tandem multiplication of the *ATM1* allele, is a cause of ketoconazole resistance in *M. restricta* KCTC 27529. In *M. restricta* KCTC 27550, genomic tandem multiplication of the *ERG11* allele appeared to be the sole cause of ketoconazole resistance.

## Discussion

Ketoconazole is effective in the treatment of seborrheic dermatitis and dandruff, which are known to be associated with *Malassezia*. Several clinical studies have demonstrated the efficacy of ketoconazole. For example, a large-scale clinical trial showed that 575 patients suffering from scalp seborrheic dermatitis and dandruff were treated with a 2% ketoconazole shampoo for 2–4 weeks, resulting in 88% improvement (Peter and Richarz-Barthauer, 1995). The same study also showed lower disease recurrence rates in the group using the shampoo containing ketoconazole than in the placebo group (Peter and Richarz-Barthauer, 1995). Furthermore, a randomized study of 66 patients with seborrheic dermatitis and dandruff compared the efficacy of shampoo containing different concentrations of ketoconazole and showed significantly decreased flakiness and *Malassezia* density in the group treated with a higher concentration of ketoconazole (Pierard-Franchimont et al., 2001). This proven effect of ketoconazole has led to its use in the treatment of skin diseases associated with *Malassezia*. However, frequent and prolonged use of ketoconazole might also cause the emergence of ketoconazole-resistant fungal strains. In the current study, we isolated ketoconazole-resistant *M. restricta* strains, KCTC 27529 and KCTC 27550; their MICs against the drug were significantly higher than that of the susceptible strain. MICs of the resistant strains against terbinafine, amphotericin B, and ZPT were similar to those of other ketoconazole-susceptible strains; thus, we considered that the drug resistance of KCTC 27529 and KCTC 27550 was specific to ketoconazole.

In the current study, we carried out comparative genome analysis to understand the mechanism of ketoconazole resistance in KCTC 27529 and KCTC 27550 and observed tandem multiplications of the genomic regions containing the genes encoding the *ATM1* and *ERG11* homologs in the genomes of the strains, respectively. Furthermore, we found that the multiplication of genes in the resistant strains resulted in significantly increased transcript levels compared with those in the susceptible reference strain KCTC 27527. In the resistant strain KCTC 27529, increased expression of *ATM1* may cause an increase in the intracellular heme contents and, in turn, increase the activity of Erg11, which contains heme as a cofactor (Balding et al., 2008; Kalb et al., 1987). Our interpretation is supported by results from previous studies showing that depletion of Atm1 in *S. cerevisiae* and *C. neoformans* resulted in decreased intracellular heme content (Hausmann et al., 2008; Do et al., 2018). Another possible explanation for the association between the increased expression of *ATM1* and ketoconazole resistance in the resistant strain KCTC 27529 is the role of Atm1 in the susceptibility of the cells to oxidative stress as shown in the current study.

Numerous studies have revealed that overexpression of *ERG11* increases the levels of the antifungal target protein and, therefore, causes resistance to azole antifungal drugs (Flowers et al., 2012; Feng et al., 2016, 2017). Moreover, a number of studies have demonstrated that overexpression is frequently induced by the increased copy number of *ERG11*, which is a result of the amplification of a segment or the entire chromosome (Marichal et al., 1997; Selmecki et al., 2008; Sionov et al., 2010; Kim et al., 2018). Studies with *C. albicans* also showed that, among fluconazole-resistant isolates, ~20% displayed segmental aneuploidies of the left arm of chromosome 5 containing *ERG11* and increased gene expression levels (Selmecki et al., 2006, 2008). Similarly, duplication of the whole chromosome 1 carrying *ERG11* and upregulation of genes in the chromosome was observed in fluconazole-resistant *C. neoformans* (Sionov et al., 2010). Previously, we studied the mechanism of ketoconazole resistance in *M. pachydermatis*, which was isolated from a dog with otitis externa. Tandem quadruplication of the ~64 kb genomic locus containing *ERG11* along with increased expression was identified in the strain. In the same study, we also observed segmental multiplication of the chromosomal region containing *ERG11* in the ketoconazole-resistant strains generated by *in vitro* evolution, suggesting that an increased copy number of *ERG11* in response to ketoconazole is a common resistance mechanism in *Malassezia* (Kim et al., 2018). Considering the above findings, increased expression of *ERG11* caused by the multiplication of the *ERG11* copy number might be one of the main causes of ketoconazole resistance in *M. restricta* KCTC 27550.

Previous studies with other azole-resistant fungi, including *S. cerevisiae, C. albicans*, and *C. neoformans*, have reported that large DNA segments ranging from segmental to the entire chromosome were amplified (Koszul et al., 2006; Selmecki et al., 2006; Sionov et al., 2010). However, we observed a tightly amplified region, including one gene in the ketoconazole-resistant *M. restricta* KCTC 27529 and KCTC 27550. Moreover, these resistant isolates were obtained from completely unrelated patients suggesting that a short segmental genomic multiplication has been evolved to develop the drug-resistant phenotype in *M. restricta*, which possesses a very compact genome.

Hyperactivation of the drug efflux pump, which exports intracellular azole, is one of the well-identified mechanisms of azole resistance (Paul and Moye-Rowley, 2014). In fluconazole-resistant *Candida species*, elevated expression of genes encoding plasma membrane efflux proteins, such as *CDR1, CDR2*, or *MDR1*, and increased efflux have been observed frequently (Leppert et al., 1990; Brun et al., 2004; Lamping et al., 2007; Berkow et al., 2015; Kim et al., 2017). Moreover, a recent study with fluconazole or voriconazole-resistant *M. furfur* and *M. pachydermatis* showed a significant decrease in the MICs of drugs in the presence of efflux protein inhibitors, indirectly suggesting the role of the drug efflux pump in azole-resistant *Malassezia* (Iatta et al., 2017).

We observed significant upregulation of MRET_2329, a homolog of *S. cerevisiae PDR5* and *C. albicans CDR1*, in the ketoconazole-resistant strain KCTC 27529 compared to the susceptible reference strain, which supported its significantly increased drug efflux phenotype. The regulatory mechanism of *PDR5* and *CDR1* expression in *S. cerevisiae* and *C. albicans* was studied; it was found that these genes are transcriptionally regulated by the transcription factors Pdr1/Pdr3 and Tac1, respectively (Katzmann et al., 1996; Coste et al., 2004). However, *M. restricta* lacks the homologs of genes encoding these transcription factors, implying that the regulatory mechanism underlying the expression of the *PDR5* homolog in this fungus might be different from those in *S. cerevisiae* and *C. albicans* and still needs to be explored. Collectively, we concluded that, in addition to multiplication in the genomic region containing *ATM1*, increased drug efflux mediated by increased expression of the Pdr5 homolog is one of the resistance mechanisms in KCTC 27529.

Overall, the results of our study suggest that multiplication of the genomic loci encoding genes involved in ergosterol synthesis, and oxidative stress response and overexpression of drug efflux protein are the mechanisms underlying ketoconazole resistance in *M. restricta*. Furthermore, our data imply that a short segmental genomic rearrangement, such as a tandem multiplication, might be a common adaptive mechanism in *Malassezia* against azole antifungal drugs.

## Data Availability Statement

The datasets generated for this study can be found in the Sequence Read Archive (SRA) database of the National Center for Biotechnology Information (NCBI) (Bioproject number, PRJNA592379).

## Author Contributions

MP and Y-JC performed the experiments. MP, Y-JC, YL, and WJ analyzed and interpreted the data and prepared the manuscript. All authors reviewed the manuscript.

## Conflict of Interest

The authors declare that the research was conducted in the absence of any commercial or financial relationships that could be construed as a potential conflict of interest.
