# Supplementary material for: Genomic Multiplication and Drug Efflux Influence Ketoconazole Resistance in Malassezia restricta
Source: Front Cell Infect Microbiol. 2020 Apr 30;10:191. doi: 10.3389/fcimb.2020.00191 (PMC7203472; doi:10.3389/fcimb.2020.00191)
Supplement: Supplementary file 1 [file Data_Sheet_1.docx]

Supplementary Material

# Supplementary Table

Supplementary Table S1. Primers used in this study.

| **Primers** |  | **Sequence (5'-3')** | **Target gene ID** |
| --- | --- | --- | --- |
| ERG11_start1 |  | ATGCACCTGGCTACGGGCCT |  |
| ERG11_start2 |  | ATGGCCTCCTTGCTGGCGAGTT |  |
| ERG11-2 |  | GCGACGGAAGTTGTTCGGTAG |  |
| ERG11-3 |  | CAAGCTGTACCACGACCTCGATG |  |
| ERG11-4 |  | CTACGTACGGGACGTAAACACAATG |  |
| TEF1p_F_XbaI |  | AGCTCCTCTAGACCACCCGTTGAATTTTTGGTGCGAAG |  |
| TEF1p_R_XhoI |  | GTCTTCCTCGAGCATTTTGAAGTTTTCTGTGGAGATCGTTAG |  |
| TEF1p_ATM1-1 |  | AGCACTCACCTCGATCCTCTTGTC |  |
| TEF1p_ATM1-2 |  | CTTCCGTGTTAATACAGATAAACCAATTTGAGTGTGGGAGGGTTTCAGAGC |  |
| TEF1p_ATM1-3 |  | CTAACGATCTCCACAGAAAACTTCAAAATGGGCTTCGGCTCCTGCAGTCGAC |  |
| TEF1p_ATM1-4 |  | TAGGTGAACTGGCGTACTTAGTTCCACG |  |
| TEF1p_ATM1-5 |  | CTCATCATCGTCGTCATCGTCATCATCC |  |
| TEF1p_ATM1-6 |  | GCCAGTTTGACGCTCCAGATGAAACTTC |  |
| qRT_MRE_ACT | Forward | CCTTCCTTGCCCTCTTCTCAT | MRET_1518 |
|  | Reverse | AGCGACGACAGGGACAATG |  |
| qRT_MRE_ERG11 | Forward | CCCCCAGGTCCAGCAACT | MRET_3233 |
|  | Reverse | CGAACCACGGTGGGATAAAT |  |
| qRT_MRE_ATM1 | Forward | CGTGTCCTCGATGTTGGATGT | MRET_4198 |
|  | Reverse | TCAGAGAAACGGGCAATTTCA |  |
| qRT_MRE_CDR1 | Forward | CCAACGCCACCCTTGTAGTC | MRET_2329 |
|  | Reverse | GCGGGTGGTCCTCTACCA |  |
| qRT_MRE_CDR2 | Forward | TCAGGCGGCCCTACCA | MRET_2330 |
|  | Reverse | GAGCGGCGTATCCGTCAA |  |
| qRT_CNEO_TEF2 | Forward | GGCGGTCCGCAAATCC | CNAG_00044 |
|  | Reverse | TGCAGATTGCTCAGATTCATAGTCT |  |
| qRT_CNEO_ATM1 | Forward | AGGAGCGACGAGCAAAGTTAGT | CNAG_04358 |
|  | Reverse | CACCTGTACCCGCCCAGAT |  |

# Supplementary Fig. S1.


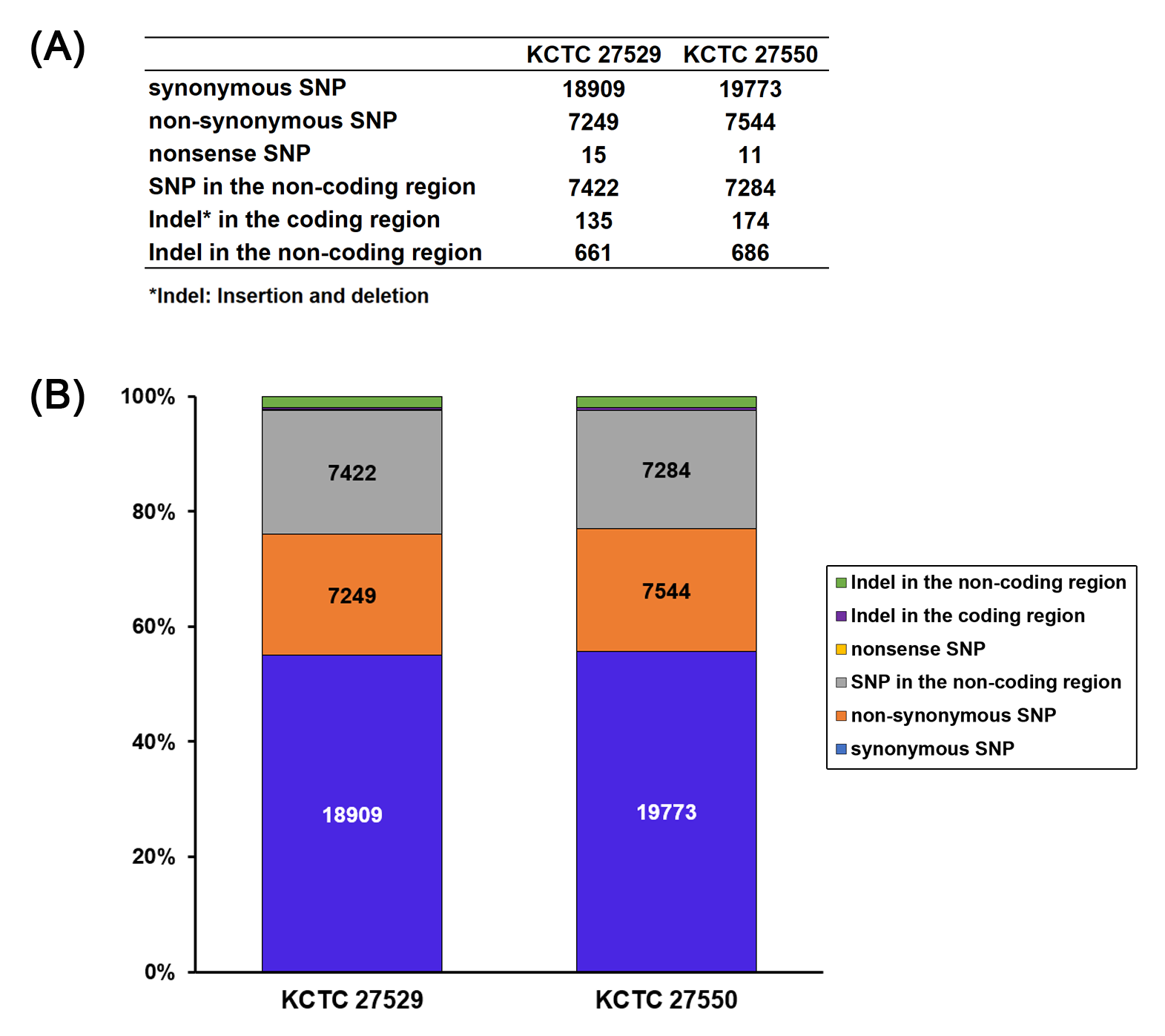


Supplementary Figure 1. The number of mutations (A) and distribution (B) in the genome of the resistant strains KCTC 27529 and KCTC 27550.
